# THE CONTRIBUTION OF COMMUNITY/NEIGHBORHOOD TO COMMON PREDICTORS OF NON-COMMUNICABLE AGING RELATED DISEASE

**DOI:** 10.1093/geroni/igae098.4084

**Published:** 2024-12-31

**Authors:** Alicia DeLouize, Paul Kowal, Nirmala Naidoo, Srean Chhim, Heng Sopheab, Josh Snodgrass, Savuth Chin

**Affiliations:** University of Oregon, Eugene, Oregon, United States; The Australian National University, Canberra, Australian Capital Territory, Australia; World Health Organization, Geneva, Geneve, Switzerland; National Institute of Public Health, Phnom Penh, Phnom Penh, Cambodia; National Institute of Public Health, Phnom Penh, Phnom Penh, Cambodia; University of Oregon, Eugene, Oregon, United States; National Institute of Public Health, Phnom Penh, Phnom Penh, Cambodia

## Abstract

Non-communicable diseases (NCDs) are now the leading causes of death globally. Their increasing rates have been associated with market integration and they tend to cluster in certain neighborhoods and communities, yet research has mostly focused on individual level causes. The aim of this research was to identify neighborhood/community level associations with NCDs that are often attributed to individual lifestyle factors. The World Health Survey Plus (WHS+) was conducted in Cambodia in 2023 and is among the first studies to collect nationally representative point-of-care biomarker data along with anthropometric, performance test, and survey data (N = 5,275). Multi-level models were run, with 98 regions and 276 communities as random intercepts, predicting rates of diabetes, hypercholesterolemia, hyperlipidemia, hypertension, arthritis, angina, stroke, and frailty level. Between 22% to 77% of the variance usually attributed to fixed effect independent variables (education, log of household income, salt, alcohol, tobacco, waist-to-stature-ratio (WSR), anxiety, stress, and sedentary behavior) was attributed to group-level fixed effects. Alcohol, sedentary behavior, stress, anxiety, WSR, and education were group-level variables that were conflated at the individual level for at least one NCD. Prevention and treatment of non-communicable diseases have mostly centered around individual lifestyle changes, yet there are likely ecological causes that can be targeted.

